# Reassessing the Use of VCUG in Pediatric UTIs: Are We Overusing an Invasive Diagnostic Tool?

**DOI:** 10.3390/healthcare13131513

**Published:** 2025-06-25

**Authors:** Ivana Fratrić, Dragana Milutinović, Maja Samardžić Lukić, Dragana Živković

**Affiliations:** 1 Faculty of Medicine, University of Novi Sad, 21000 Novi Sad, Serbia; maja.samardzic-lukic@mf.uns.ac.rs (M.S.L.); dragana.zivkovic@mf.uns.ac.rs (D.Ž.); 2 The Institute for Children and Youth Health Care of Vojvodina, 21000 Novi Sad, Serbia

**Keywords:** voiding cystourethrography, vesicoureteral reflux, pediatric urology, pediatric nephrology, urinary tract infection, diagnostic imaging, radiation exposure

## Abstract

**Background/Objective**: Voiding cystourethrography is the gold-standard diagnostic tool for detecting vesicoureteral reflux and is commonly requested by pediatricians, pediatric nephrologists, emergency pediatricians, and pediatric urologists. However, VCUG is invasive, exposes patients to radiation, and carries a risk of iatrogenic urinary tract infection (UTI). This study aimed to assess the correlation between VCUG findings and factors such as age, gender, referring specialist, and clinical indication for the procedure to identify opportunities to reduce unnecessary VCUG examinations. **Methods**: A retrospective analysis of 197 pediatric patients who underwent VCUG over 12 months at the Institute for Child and Youth Health Care of Vojvodina was conducted. **Results:** The Mann–Whitney U test showed no statistically significant age difference between patients with normal (median: 2.5 years) and pathological (median: 3 years) VCUG findings (Z = −0.415, *p* = 0.678). The chi-square test showed that patients with a single urinary tract infection (10 patients) and other clinical indications (24 patients) had a higher chance of normal VCUG findings (0.041 and 0.011, respectively). Binary logistic regression analysis showed that patients referred by pediatric urologists were 2.06 times more likely to have pathological VCUG findings than those referred by pediatric nephrologists (*p* = 0.013, OR = 2.059; 95%CI: 0.166–3.634). Regarding clinical indications, the chance that VCUG findings would be normal was 2.7 times higher in patients with other indications than in patients with recurrent UTIs (*p* = 0.038, OR = 2.729; 95% CI: 1.055–7.059). **Conclusions**: Pediatric urologists tend to refer patients for VCUG more selectively than pediatric nephrologists. Avoiding VCUG in cases of a single UTI or non-specific clinical indications could significantly reduce the number of unnecessary procedures, minimizing patient exposure to radiation and potential complications.

## 1. Introduction

Over the past two to three decades, voiding cystourethrography (VCUG) was historically indicated for all pediatric patients with urinary tract infections (UTIs). At that time, it was believed that complications such as pyelonephritis and renal impairment were attributable to vesicoureteral reflux (VUR) of any severity [1]. Current understanding, however, recognizes that most VUR cases resolve spontaneously [2], prompting a re-evaluation of the VCUG examination protocol.

The clinical necessity of performing a VCUG following a singular episode of UTI remains a contentious issue. The primary objective of the VCUG is to identify high-grade VUR before the onset of renal damage, thereby mitigating the risk of adverse long-term outcomes in affected patients. Variability in VCUG protocols across different medical facilities complicates comparisons of outcomes and hinders the derivation of conclusive findings. VCUG results provide critical insights that guide clinical decisions regarding the continuation of conservative management or the need for surgical intervention, necessitating diligent follow-up in both scenarios [3].

As the primary diagnostic tool for detecting VUR, VCUG is regarded as the “gold standard” and can be ordered by various specialists, including pediatricians, pediatric nephrologists, emergency room pediatricians, and pediatric urologists. Nonetheless, the procedure is associated with certain disadvantages, including its invasive nature, exposure to ionizing radiation, and the potential risk of iatrogenic UTI [1,3].

Considering these factors and the likelihood of identifying a benign condition, it has been proposed that VCUG should be reserved for carefully selected cases rather than used as a routine diagnostic protocol. In this context, we hypothesize that certain patient and referral characteristics, such as a single UTI, male gender, and referral by non-urology specialists, have a lower likelihood of pathological VCUG findings and may reflect potential overuse of the procedure.

## 2. Materials and Methods

### 2.1. Study Design and Setting

A retrospective analysis of pediatric patients who underwent VCUG over 12 months at the Institute for Child and Youth Health Care of Vojvodina was conducted.

### 2.2. Sample and Data Collection

Clinical data of all patients aged 0 to 18 who underwent VCUG over 12 months were collected and analyzed. Data included the following: patient age, gender, referring specialist—pediatric urologist or pediatric nephrologist, and indication for UCUG.

All patients underwent standard procedures according to their age while performing VCUG. All follow-up patients continued to be monitored by the same doctor (pediatric urologist or pediatric nephrologist) who initially indicated VCUG and every other VCUG. The clinical indications for VCUG were the following: UTIs, recurrent UTIs, hydronephrosis, follow-up, and others. UTI diagnosis required pyuria and a positive urine culture. Recurrent UTI was defined as repeated UTI with pyuria and a positive urine culture. Other patients included preparation for transplantation and dysfunctional voiding.

### 2.3. Data Analysis

Collected data were analyzed statistically using IBM SPSS 23 Statistics, while the statistically significant results were determined at *p* < 0.05. Descriptive methods were used to determine the number (n) and percent (%) of categorical variables. The patients’ age was presented using a diagram with median and interquartile range (25th–75th percentile) because the Shapiro–Wilks test showed that the population is not normally distributed (*p* = 0.000). The chi-square and Fisher’s exact tests were used to test the difference between categorical variables, while the Mann–Whitney U test was used for the age difference. Multiple logistic regression analysis was used to analyze the possible combined effects of predictors on VCUG findings. Multicollinearity among predictors was assessed using linear regression and Variance Inflation Factor (VIF) analysis. The Hosmer–Lemeshow test was applied to evaluate the goodness of fit of the logistic regression model.

### 2.4. Ethical Considerations

The study was conducted following ethical guidelines and legal standards. It adhered to the principles outlined in the Declaration of Helsinki and was approved by the Faculty of Medicine Commission for the Ethics of Clinical Research, the University of Novi Sad, Serbia, 01-39/239/1 of 9 September 2022.

## 3. Results

A total of 197 VCUG examinations were performed over 12 months (from October 2022 to September 2023). A statistically significant difference was found when comparing the results of the VCUG (pathologic vs. normal) concerning the referring specialists (χ^2^ = 5.578; df = 1; *p* = 0.018) and some clinical indications for the VCUG (follow-up, single UTI, and other indications). At the same time, there was no statistically significant difference in gender (χ^2^ = 0.016; df = 1; *p* = 0.900) (Table 1).

Normal findings on VCUG were more frequently found in male patients and those referred by pediatric nephrologists, but there was no statistically significant difference. The most common clinical indication for VCUG was recurrent UTI, followed by hydronephrosis, but without a statistically significant difference between the groups. Patients whom the pediatric urologist referred had pathological findings on VCUG more frequently (χ^2^ = 4.167; df = 1; *p* = 0.041) (Table 1).

Another statistically significant difference was found in the number of follow-up VCUG patients. As expected, these children more often had pathological findings on VCUG (χ^2^ = 26.273; df = 1; *p* = 0.000). In contrast, children with single UTI (χ^2^ = 6.400; df = 1; *p* = 0.011) and other indications for VCUG (χ^2^ = 4.167; df = 1; *p* = 0.041) were more likely to have normal findings on VCUG (Table 1).

**Figure 1:**
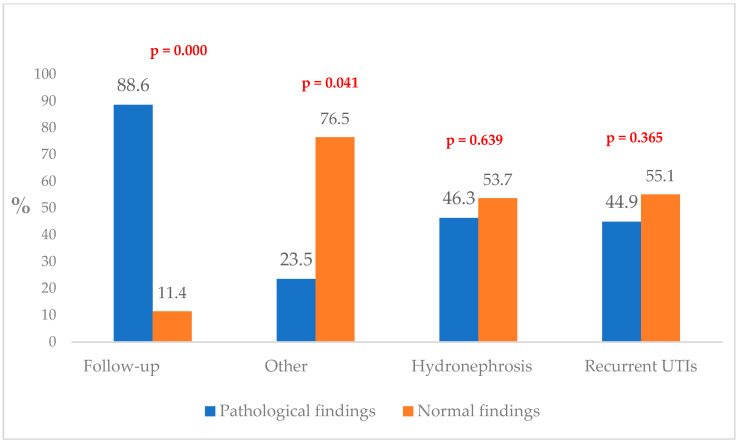
Distribution of patients by VCUG findings (normal vs. pathological) across clinical indications.

Figure 1 illustrates the distribution of patients with normal and pathological VCUG findings according to clinical indications.

All patients from this study were between 0 and 17 years old, with a positive asymmetry indicating a larger number of younger children in both groups. Four atypically older children were found in the group with pathological findings on VCUG, while in the group with normal VCUG findings, there were two older children. The median patient age in the group with pathological findings on VCUG was 3 years, and 2.5 years in the group with normal findings on VCUG. In both groups, the interquartile range was 6 years (1–7 years), meaning that 25% of children were younger than 1 year, and 50% were between 1 and 7 years old. Among the children with pathological findings, four were of atypical age (15–17 years old), while among those with normal findings, two were of atypical age (17 years). The results of the Mann–Whitney U test did not show a statistically significant age difference between patients with normal findings and those with pathological findings on VCUG (Z = −0.415; *p* = 0.678).

When we excluded 44 follow-up VCUG patients (22.3%), 153 patients (77.7%) remained who had some indications and who we analyzed further. In patients who had an indication, there was no statistically significant difference between VCUG findings and indications (χ^2^ = 6.338; df = 3; *p* = 0.096), gender (χ^2^ = 1.709; df = 1; *p* = 0.185), and referring specialist (χ^2^ = 0.004; df = 1; *p* = 0.865).

Table 2 shows that the normal finding was statistically significantly more frequent in male patients (χ^2^ = 7.353; df = 1; *p* = 0.007), those with one UTI (χ^2^ = 6.400; df = 1; *p* = 0.011), and in patients with some other indications (χ^2^ = 4.167; df = 1; *p* = 0.041).

The results of the chi-square test of independence indicated a statistically significant association between gender and indications (χ^2^ = 22.127; df = 2; *p* = 0.000), as well as between indications and VCUG findings (χ^2^ = 36.739; df = 2; *p* = 0.000).

The partial effect of gender, age, referring specialist, and clinical indication on the outcome of pathological findings presented in Table 3 indicates that patients referred by pediatric urologists were 2.06 times more likely to have pathological findings of VCUG than those referred by pediatric nephrologists (*p* = 0.013, OR = 2.059; 95%CI: 0.166–3.634). Indications significantly influence the pathological findings (*p* = 0.000, OR = 2.012; 95%CI: 1.525–2.654). Gender and age did not affect VCUG findings.

Table 4 shows the results of a multivariate logistic regression analysis of the simultaneous influence of gender, age, and clinical indication on the normal finding, with recurrent UTI as a reference value.

Collinearity between the tested predictors was checked by linear regression and VIF analysis. All VIF values are below 1.18, meaning no significant collinearity exists between the variables (gender, age and indication). From the aspect of collinearity, the model is stable and valid. The Hosmer-Lemesh test shows a good fit of the model (χ^2^ = 10.041; df = 8; *p* = 0.262).

Gender and age did not have a statistically significant effect on VCUG findings (*p* < 0.05). Regarding clinical indications, the chance that VCUG findings would be normal was 2.7 times higher in patients with other indications than in patients with recurrent UTIs (*p* = 0.038; OR = 2.729; 95% CI: 1.055–7.059). The likelihood that the VCUG findings would be normal in the follow-up patients was 89.5% lower than in patients with recurrent UTI (*p* = 0.000; OR = 0.105; 95% CI: 0.037–0.296) (Table 4).

The multivariate logistic regression analysis in Table 5 shows the simultaneous influence of age, gender and indication without follow-up patients on normal findings, where recurrent UTIs are the reference value. VIF values < 1.25 indicate the absence of significant collinearity among the variables (gender, age and indication), which means the model is stable and valid. The Hosmer-Lemesh test shows a good fit of the model (χ^2^ = 12.453; df = 8; *p* = 0.132).

Gender and age are not statistically significant predictors in the multiple model (*p* < 0.05). The chance that VCUG findings would be normal was 3.1 times higher in patients with other clinical indications than in patients with recurrent UTIs (*p* = 0.024; OR = 3.067; 95% CI: 1.159–8.115) (Table 5).

## 4. Discussion

VCUG is generally indicated in pediatric cases where there is a suspicion of VUR, typically following episodes of UTI. This study supports the utilization of VCUG as a diagnostic tool after repeated UTIs, but not after a single UTI.

Historically, it was believed that VUR was a precursor to renal damage in pediatric populations [1]. Recent evidence, however, suggests that VUR does not invariably predict future renal impairment [4]. A multicenter, randomized controlled trial conducted by Garin [5] failed to demonstrate sufficient evidence to support the use of antimicrobial prophylaxis in children with VUR, a finding echoed in research by Hari et al. [6] and Pennesi et al. [7].

The difference between pediatric urologists and pediatric nephrologists in referring children for VCUGs largely stems from their training focus and clinical priorities. The focus of the pediatric urologists is on surgical and structural abnormalities such as VUR, posterior urethral valves, and obstruction. They are familiar with structural imaging; they often use VCUG, ultrasound, and DMSA scans, which implies a more accurate referral diagnosis.

Given that a majority of children present with normal VCUG findings following a single UTI, this raises the following question: Is this invasive procedure, which is associated with radiation exposure and the potential for iatrogenic UTIs, warranted for all cases? This practice commenced in the late 20th century when guidelines advocated for renal and bladder ultrasound following a single febrile UTI [1]. Subsequently, twelve years later, VCUG was recommended for children under the age of 2 following febrile UTI only if ultrasound indicated hydronephrosis or other findings suggestive of high-grade VUR, according to revised guidelines published by the American Academy of Pediatrics (AAP) [8,9]. Consequently, the indication for VCUG has been reduced in the context of febrile UTIs; however, this recommendation has not been uniformly adopted across various medical institutions. Some facilities continue to perform VCUG after a single instance of UTI, particularly in male patients [3]. The AAP guidelines published in 2011 recommend VCUG following a second UTI or if abnormalities are detected on RBUS [8]. In contrast, the European Association of Urology (EAU) advises VCUG if renal scarring is identified on dimercaptosuccinic acid (DMSA) scanning [10]. The National Institute for Health and Care Excellence (NICE) guidelines differ across age groups and are contingent upon the type of UTI, suggesting VCUG after atypical or recurrent UTIs in children under six months of age [11] (Table 6).

VCUG should not be routinely used in children between 6 months and 3 years but should be considered when dilatation is detected on the ultrasound, when there is poor urine flow, non-E. coli infection, or a family history of VUR according to NICE guidelines [11].

Contrast-enhanced voiding urosonography (ceVUS) has emerged as a promising non-invasive alternative to traditional VCUG for diagnosing and evaluating VUR in pediatric patients [12]. Its advantages are the absence of ionizing radiation, better patient comfort, and the ability to provide real-time dynamic urinary tract imaging during voiding. Microbubble contrast agents used in ceVUS allow high-resolution imaging of the bladder and ureters, offering a clear visualization of reflux episodes without the need for invasive catheterization or radiation exposure. However, this technique requires trained personnel and specialized equipment. On the other hand, it has further advantages such as reducing healthcare costs, especially in children who require follow-up visits. According to the meta-analysis conducted in 2022. by Yousefifard et al. ceVUS carries a risk of false-negative findings in 3% of cases [13].

Healthcare professionals responsible for indicating and conducting VCUG should remain informed about the inconsistencies present in the existing literature [14]. Before ordering this diagnostic test, clinicians must carefully weigh its associated risks and benefits. The advantages of VCUG include elucidating the etiology of the UTI, confirming the presence and degree of VUR, and determining its laterality, which can inform management decisions such as watchful waiting, antibiotic prophylaxis, endoscopic intervention, or surgical ureteral reimplantation. Ultimately, VCUG has the potential to prevent recurrent pyelonephritis and its associated complications. However, most children with UTIs referred for VCUG are likely to exhibit low-grade VUR with a significant rate of spontaneous resolution or present with normal findings, prompting the consideration that administering VCUG after a single UTI may constitute an overdiagnosis. Future research after introducing ceVUS is needed to identify its accuracy in detecting VUR and potential changes to reduce false negative findings associated with the ceVUS technique.

### Limitations

This study analyzed only conventional VCUG, as our experience with the ceVUS is still limited. We have adopted the AUA treatment protocol amended in 2017 [15], but despite that, our impression is that different personal and professional factors influence the interpretation of this and any other protocol by physicians. Therefore, we wanted to analyze the indications for VCUG at our Institute. Further research is required following the introduction of ceVUS in our Institute, as it may reduce the reliance on VCUG.

## 5. Conclusions

The findings of this study revealed that patients referred for VCUG by a pediatric urologist who subsequently attended a follow-up VCUG may have a higher likelihood of experiencing pathological findings on the VCUG compared to patients who have a history of a single UTI or other indications for undergoing the procedure. Additional research is warranted to explore strategies for reducing the incidence of unnecessary VCUG examinations. The study implies that an internal protocol could be proposed to limit the overdiagnosis after a single UTI, as well as introduce ceVUS as a standardized procedure at our Institute.

## Figures and Tables

**Table 1:** Descriptive values for gender, referring specialist, and clinical indications for VCUG based on the pathological or normal VCUG results and in the whole sample.

	Pathological Findings on VCUG (*n* = 101)	Normal Findings on VCUG (*n* = 96)	Total (N = 197)	χ^2^	*p*
	*n* (%)	*n* (%)	*n* (%)		
Gender ^$^				0.016	0.900
Male ^#^	44 (43.6)	40 (58.3)	84 (42.6)	0.190	0.663
Female	57 (56.4)	56 (41.7)	113 (57.4)	0.009	0.925
Referring specialist ^$^				5.578	0.018
Pediatric urologist ^#^	58 (57.4)	38 (39.6)	96 (48.7)	4.167	0.041
Pediatric nephrologist	43 (42.6)	58 (60.4)	101 (51.3)	2.228	0.136
Clinical indications ^$^				37.777	<0.001
Follow-up ^#^	39 (38.6)	5 (5.2)	44 (22.3)	26.273	0.000
UTIs	1 (1.0)	9 (9.4)	10 (5.1)	6.400	0.011
Recurrent UTIs	35 (34.7)	43 (44.8)	78 (39.6)	0.821	0.365
Hydronephrosis	19 (18.8)	22 (22.9)	41 (20.8)	0.220	0.639
Other	7 (6.9)	17 (17.7)	24 (12.2)	4.167	0.041

^$^ Chi-square test of independence; # chi-square goodness-of-fit test.

**Table 2:** Descriptive values for gender, referring specialist, and clinical indications for VCUG in the total sample without follow-up VCUG patients.

	Pathological Findings on VCUG (*n* = 62)	Normal Findings on VCUG (*n* = 91)	Total (N = 153)	χ^2^	*p*
	*n* (%)	*n* (%)	*n* (%)		
Gender ^$^				1.709	0.185
Male ^#^	30 (51.6)	55 (60.4)	85 (55.6)	7.353	0.007
Female	32 (48.4)	36 (39.6)	68 (44.4)	0.235	0.628
Referring specialist ^$^				0.004	0.865
Pediatric urologist ^#^	22 (35.5)	34 (37.4)	56 (36.6)	2.571	0.109
Pediatric nephrologist	40 (64.5)	57 (62.6)	97 (63.4)	2.979	0.084
Clinical indications ^$^				6.338	0.096
UTIs ^#^	1 (1.6)	9 (9.9)	10 (5.1)	6.400	0.011
Recurrent UTIs	35 (56.5)	43 (47.3)	78 (39.6)	0.821	0.365
Hydronephrosis	19 (30.6)	22 (24.2)	41 (20.8)	0.220	0.639
Other	7 (11.3)	17 (18.7)	24 (12.2)	4.167	0.041

^$^ Chi-square test of independence; # chi-square goodness of fit test.

**Table 3:** Binary logistic regression analysis of the effect of gender, age and referring specialist on the pathological findings on VCUG (N = 197).

	B	Wald	*p*	OR	95% C.I. for OR	
					Lower	Upper
Gender (Female)	0.078	0.072	0.788	1.081	0614	1.902
Age	0.021	0.397	0.529	1.022	0.956	1.092
Referring specialist (Pediatric nephrologist)	0.772	6.203	0.013	2.059	1.166	3.634
Clinical indication (Recurrent UTIs)	0.699	24.412	0.000	2.012	1.525	2.654

B: regression coefficient; Wald-Wald statistic; *p*: statistical significance; OR: odds ratio; CI: confidence interval.

**Table 4:** Multivariate logistic regression analysis of the influence of gender, age and clinical indications on a normal finding on VCUG (N = 197).

	B	Wald	*p*	OR	95% C.I. for OR	
					Lower	Upper
Gender (Female)	−0.243	0.429	0.513	0.784	0.379	1.623
Age years months	0.009	0.041	0.839	1.009	0.929	1.095
Indication (Follow-up)	−2.253	18.141	0.000	0.105	0.037	0.296
Indication (Other)	1.004	4.285	0.038	2.729	1.055	7.059
Indication (Hydronephrosis)	0.064	0.023	0.879	1.066	0.470	2.420
Constant	0.239	0.529	0.467	1.270		

B: regression coefficient; Wald-Wald statistic; *p*: statistical significance; OR: odds ratio; CI: confidence interval.

**Table 5:** Multivariate logistic regression analysis of the influence of gender, age and clinical indications without follow-up patients on a normal finding on VCUG (*n* = 153).

	B	Wald	*p*	OR	95% C.I. for OR	
					Lower	Upper
Gender (Female)	−0.617	2.397	0.122	0.539	0.247	1.178
Age years months	−0.004	0.008	0.927	0.996	0.915	1.084
Indication (Other)	1.121	5.095	0.024	3.067	1.159	8.115
Indication (Hydronephrosis)	0.214	0.251	0.616	1.238	0.537	2.857
Constant	0.399	1.393	0.238	1.490		

B: regression coefficient; Wald-Wald statistic; *p*: statistical significance; OR: odds ratio; CI: confidence interval.

**Table 6:** Current VCUG recommendations in different guidelines.

Guideline	Routine VCUG	Indications for VCUG	Rationale
AAP	No	Abnormal ultrasound, atypical cases, recurrent UTIs	Avoid unnecessary procedures; most children do not have high-grade VUR
AUA	No	Similar to AAP	Aligns with AAP approach
NICE	No	Children < 6 months, atypical/recurrent UTI	Focus on higher-risk patients
EAU	Yes	Children < 2 years, abnormal ultrasound, atypical/complex cases	Early identification of high-grade VUR to prevent renal scarring

## Data Availability

The data supporting this study’s findings are available on request from the corresponding author.

## Abbreviations

The following abbreviations are used in this manuscript:

VCUG	Voiding cystourethrography
VUR	Vesicoureteral reflux
VIF	Variance Inflation Factor (VIF)
UTI	Urinary tract infections
AAP	Academy of Pediatrics
EAU	The European Association of Urology
DMSA	Dimercaptosuccinic acid
NICE	The National Institute for Health and Care Excellence
ceVUS	Contrast-enhanced voiding urosonography
